# Unlocking the Contradictory Outcomes of Presenteeism Through a Temporal Model: Effort Exertion as a Mediator

**DOI:** 10.3389/fpsyg.2021.740411

**Published:** 2021-11-25

**Authors:** Chun-Yi Chou, Merce Mach

**Affiliations:** ^1^Department of International Business, Feng Chia University, Taichung, Taiwan; ^2^Department of Business, University of Barcelona, Barcelona, Spain

**Keywords:** presenteeism, effort exertion, job performance, work engagement, emotional exhaustion

## Abstract

The effects of presenteeism, that is, working while ill or exhausted, are unclear, as previous research has yielded contradictory results. The aim of this study was thus two-fold: clarify the differential effects of short versus long-term presenteeism and corroborate the mediating effect of effort exertion on the relationship between presenteeism and work-related outcomes. We adopt a three-wave panel design and measure all the variables at three different points (initially, after one week and after one year) to understand the effects of presenteeism over time. Our sample consists of 361 Chinese employees working in diverse industries in Taiwan. We analyze the panel data using structural equation modeling and bootstrapping. Our results reveal that presenteeism is positively associated with increased effort, work engagement, and job performance after one week. By contrast, presenteeism is negatively associated with job performance and work engagement though positively associated with emotional exhaustion after one-year. Our research contributes to clarify paradoxical results regarding presenteeism’s consequences, as well as corroborating that effort exertion mediates the relationship between presenteeism and work outcomes. We also identify practical implications for organizations managing employees working remotely, a more common reality with the outbreak of the COVID-19 pandemic, the ensuing lockdowns and digitalization which has started to become the norm for a significant proportion of working sectors. Finally, we suggest recommendations for future research on presenteeism.

## Introduction

Presenteeism, defined as working while ill (Johns, 2010), has a wide range of consequences for people and organizations; however, research regarding presenteeism in various disciplines has yielded inconsistent results (Ruhle et al., 2020). Longitudinal research conducted over one year has revealed a strong, negative relationship between presenteeism and personal well-being (Skagen and Collins, 2016) as well as job performance (Leijten et al., 2014). However, a systematic review of cross-sectional presenteeism studies revealed negligible or nonsignificant relationships between presenteeism and performance ratings (Lohaus and Habermann, 2019). Research over shorter intervals has also revealed positive relationships between presenteeism and job performance (3-month interval; Lu et al., 2013) and mental health (2-month interval; Lu et al., 2014).

These inconclusive results in presenteeism research call for further exploration on the consequences of presenteeism over time (Skagen and Collins, 2016). Furthermore, a theory that could potentially explain the uneven findings when considering short or long-term presenteeism research is missing. In recent theoretical contributions, presenteeism is viewed as a neutral act, without positive or negative valence (Ruhle et al., 2020). Therefore, this research aims to shed light on these contradictory results by applying the cognitive activation theory of stress (CATS; Ursin and Eriksen, 2004) and the conservation of resources theory (COR; Hobfoll and Wells, 1998) and by reviewing empirical evidence regarding presenteeism behavior over two time spans (one week and one year). We thus aim to achieve a more nuanced understanding of “bad presenteeism” (Lu et al., 2013; Cooper and Lu, 2016).

**Figure 1:**
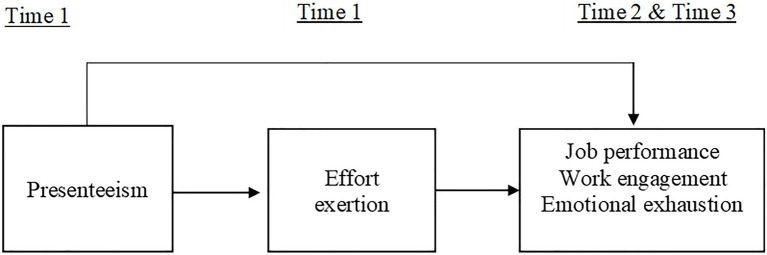
Conceptual research model.

Research regarding the effects of presenteeism has primarily focused on predicting work-related outcomes, such as job performance and emotional exhaustion (Skagen and Collins, 2016). These outcomes are thought to be influenced by “effort exertion.” Hockey (1993) highlighted the concept of effort exertion which other research has applied to link work behaviors to related outcomes (Brown and Leigh, 1996; Hockey, 1997). Overall, there are few studies in the organizational behavior field regarding effort exertion mechanisms (Yeo and Neal, 2004). The effects of the effort exerted at work and the time over which such effort is exerted are often overlooked when describing personal and organizational outcomes. Therefore, drawing on CATS and COR theories, we predict that effort exertion at work is a key underlying psychological mechanism to understand whether presenteeism leads to two distinct circumstances, namely, increased or decreased performance over different time spans. Figure 1 depicts our conceptual research model.

This research makes three valuable contributions to the presenteeism field. First, based on the CATS and the COR theories and our multiple follow-up research design, we shed light on the conflicting results regarding presenteeism and we hypothesize and test a temporal presenteeism model with short and long-term effects (one week and one year) on personal well-being and organizational effectiveness. Second, by incorporating the effort exertion construct as a key mediator, we establish a process model of presenteeism – effort exertion – health/behavioral outcomes to increase our understanding of how the neutral act of presenteeism is translated into future outcomes over various timespans. Finally, presenteeism behavior, although widely scrutinized in Western literature, is considered a key virtue in the Confucian cultural context (Chinese Culture Connection, 1987), emphasizing values such as hard work, diligence, and perseverance (Bond, 1988). The Chinese work culture is thus an ideal context to examine the generalizability of Western presenteeism research and to further explore the psychological mechanisms that explain the paradoxical short and long-term outcomes of presenteeism.

## Hypotheses Development

### Contrasting Outcomes of Short and Long-Term Presenteeism

Inspired by a recent review across disciplines (Ruhle et al., 2020) that cautioned against overgeneralizing the negative effects of presenteeism behavior, we considered presenteeism to be an act without positive or negative valence. We thus avoided obscuring the possible positive effects of this behavior. Although presenteeism has long been conceived as a counterproductive work behavior (Aronsson and Gustafsson, 2005) that reduces employee well-being and organizational effectiveness over the long term, for example, 1.5years (Demerouti et al., 2009) and 2years (Conway et al., 2014; Gustafsson and Marklund, 2014), recent research has suggested that the negative effects of presenteeism in terms of productivity loss and on personal and organizational outcomes have been overstated (Johns, 2012; Cooper and Lu, 2019; Ruhle et al., 2020). For example, Lu et al. (2014) found no lasting effects of presenteeism on mental health, physical health, or burnout over a two-month interval. Another study adopted a three-month interval and found no evidence of long-lasting or damaging effects on productivity or job performance (Lu et al., 2013). Due to these inconclusive findings, recent research has suggested that presenteeism can also be considered an adaptive coping strategy for individuals and may increase short-term job performance without damaging personal health (Karanika-Murray and Biron, 2020).

We propose that the length of time that workers adopt presenteeism behavior should be considered to examine its effects on personal well-being and organizational effectiveness. This approach is consistent with Skagen and Collins’ suggestion (2016) that future research on presenteeism should consider timeframes to understand the long-term consequences of presenteeism. Thus, to help explain the different outcomes of presenteeism over different time spans, we apply CATS theory (Ursin and Eriksen, 2004).

CATS (Ursin and Eriksen, 2004) is a general and comprehensive theory of stress that is attuned with contemporary stress theories such as the job demand-control model (Karasek, 1979) and the effort-reward imbalance model (Peter and Siegrist, 1997). CATS explains the effects of the expectation of stress in determining personal and organizational outcomes; these expectations are demand-control (resource) expectancy and effort-reward expectancy. CATS also incorporates a time perspective and explains the effects of stressors over different time intervals by including the time expectancy of a stressor. In fact, the basic assumptions of CATS theory (Ursin and Eriksen, 2004) are that stressors are normal and healthy stimuli and that the stress response is necessary. Corroborating work by Hockey (1993, 1997), which highlights the importance of considering the biological context of behaviors in explaining human behavior, CATS theory maintains that, if an individual expects a stressor will be resolved within a short time, the stress response is simply an increase in stimulation without any detrimental health effects. However, if stressors persist for longer than the expected time, stress can contribute to negative health outcomes.

In line with the CATS framework, we propose that individuals going to work in suboptimal physical conditions within a limited and predictable timespan (e.g., short-term to meet a project deadline) will allocate more energetic resources such as effort to meet goals and that this may not have any negative effects on those individuals; in addition, such additional effort will facilitate the attainment of performance goals (Hockey, 1997). Thus, we propose that, over a one-week interval (denoted as Time 2 [T2]), presenteeism behavior has positive cross-lagged effects on job performance and work engagement but does not increase emotional exhaustion (Hypothesis 1, H1).

However, considering the time expectation component of CATS theory, routinely carrying out excessive work with no clear end date is likely to have negative effects because sustained stimulation due to continual overwork causes sensitization and extensive activation of the psychobiological system, leading to negative health outcomes. Also, recruiting the energetic resources to meet chronic work demands has psychological and physiological costs (Hockey, 1997). This reasoning is also corroborated by COR theory (Hobfoll, 2001), suggesting that stress will result from a loss or threat of losing resources or due to an imbalance between invested resources and expected returns (Halbesleben, 2006), especially in long-term scenarios (Hobfoll et al., 2018).

When individuals experience ongoing losses or expect substantial resource losses, they suffer from resource depletion, which ultimately leads to exhaustion or dysfunctional behavior (Hobfoll et al., 2018). We considered stress over time by examining three intervals: T1, the initial assessment; T2, one week after T1; and T3, one year after T1. Accordingly, we propose the following cross-lagged hypotheses:

*H1*: Presenteeism at T1 has positive cross-lagged effects on a) job performance and b) work engagement at T2.

*H2*: Presenteeism at T1 has a) negative cross-lagged effects on job performance, b) work engagement at T3, and c) positive cross-lagged effects on emotional exhaustion at T3.

### Effort Exertion as a Mediating Mechanism for the Outcomes of Presenteeism

Research regarding the consequences of presenteeism has primarily concentrated on assessing the predictive strength of presenteeism measures for outcomes (see the systematic review by Skagen and Collins, 2016, or the meta-analysis by Lohaus and Habermann, 2019). Nevertheless, previous studies have ignored the underlying mediating mechanisms that translate presenteeism into outcomes and thus connect behaviors with their effects. Failing to do so prevents studying the consequences of going to work while feeling unwell, something which has yielded inconsistent findings regarding the outcomes of presenteeism behavior (e.g., positive well-being and performance; Lohaus et al., 2020) and negative well-being and productivity (Warren et al., 2011). Understanding presenteeism’s underlying psychological mechanisms is warranted for both extant literature and practical implications.

Effort has been recognized as an important mechanism in translating inputs into outputs in the organizational setting, especially under high work stress circumstances (Hockey, 1997). Effort exertion is the direction, intensity, and persistence of the effort a person applies to execute a chosen behavior (De Cooman et al., 2009). Researchers have thus far conceived displaying presenteeism behavior as comparable to exerting intensive effort. Specifically, working through illness instead of taking sick leave has been seen as the amount of effort expended in work-related tasks (Miraglia and Johns, 2016). However, overt behaviors like presenteeism may require or encourage employees to dedicate extra effort to work, though they are distinct concepts. Although presenteeism can result in dedicated effort, people with a suboptimal physiological condition may be psychologically absent and thus exert limited effort while working. Unlike the observable act of presenteeism, effort exertion is not immediately visible (Kanfer, 1990). Therefore, presenteeism and effort exertion are distinct concepts and must be refined in order to explain presenteeism’s conflicting outcomes.

By contrast, the limited cross-sectional research dedicated to effort exertion in the work setting has yielded consistent conclusions. Results have demonstrated that effort exertion is an underlying mechanism for translating work motivation into job performance and satisfaction (Dysvik and Kuvaas, 2013). Therefore, greater effort exertion leads to increased job performance (Brown and Peterson, 1994; Brown and Leigh, 1996). Although effort exertion is a key mechanism, empirical research is scarce, and existing theory does not clearly describe the links between individuals, work characteristics, and outcomes (Yeo and Neal, 2004). Accordingly, we apply the CATS and COR frameworks to examine the mediating effects of effort exertion on the relationship between overt presenteeism behavior and short- and long-term work-related outcomes.

To maintain the desirable performance, individuals will mobilize the resources they can apply to perform their professional tasks (Hockey, 1997). In the case of this study, employees who go to work while feeling ill will navigate more effort to compensate for their suboptimal situation and achieve the expected performance. Building on CATS principles, we contend that individuals who go to work while feeling ill for a short and expected period of time (e.g., a few days) to achieve their work goals will allocate their energetic resources to push themselves and exert sufficient effort to complete the necessary tasks (Hockey, 1997). Over these short intervals, intensified effort can result in achieving a performance goal without negative health effects, although it produces emotional stimulation (Ursin and Eriksen, 2004). Based on CATS theory (Ursin and Eriksen, 2004), we predict that, in the short-term, presenteeism is indicative of individual effort and thus facilitates performance and engagement without causing exhaustion (H3).

Considering the long-term effects of presenteeism and based on COR theory (Hobfoll, 2001), we contend that continuous effort exertion to meet work demands in a resource-loss situation diminishes personal resources and, in turn, leads to poor personal and organizational outcomes (Hobfoll et al., 2018). COR theory postulates that individuals confronted with resource-depleting circumstances adopt behaviors to preserve their remaining resources. Research has consistently shown that resource-depleting scenarios (such as working long hours or working through illness) cause further losses such as poor long-term performance and engagement (Lu and Chou, 2020). Employees who are chronically subject to these resource-depleting circumstances, although they exert effort and allocate their limited resources to their work, still prioritize retaining their personal resources to compensate for their suboptimal situations. However, applying limited energy resources for constant overwork while feeling ill or exhausted not only negatively affects physiological and psychological functions but also personal well-being (Skagen and Collins, 2016). Accordingly, people with diminished resources experience high levels of emotional exhaustion (McGregor et al., 2016) and are likely to have difficulties in continuing to exert additional efforts at work, stay engaged, and maintain high levels of performance (H4). Consequently, we propose the following hypotheses:

*H3*: Presenteeism at T1 has positive cross-lagged effects on a) job performance and b) engagement at T2 *via* effort exertion at T1.

*H4*: Presenteeism at T1 has a) negative cross-lagged effects on job performance, b) work engagement at T3 *via* effort exertion at T1, and c) positive cross-lagged effects on emotional exhaustion at T3.

## Methods

### Procedure and Participants

We first examined previous research to decide on the appropriate intervals to contrast the short-lived versus long-lasting effect of adopting presenteeism behaviors (e.g., Lu et al., 2014, for the short-term; and Leijten et al., 2014, for the long-term). We employed a three-wave panel study design in which we measured all the variables three times: initially (T1), at one week (T2), and after one year (T3).

We used a snowball sampling approach to recruit participants across different industries, occupations, organizations, and locations in Taiwan. We began the data collection process in January 2020 and finalized in January 2021. We asked students enrolled in the executive MBA at two large universities in Taiwan, students who usually have professional experience and hold managerial positions in their organizations, to help advertise the study and invite participants to take part *via* Line, a freeware app for instant communication widely used in Taiwan. To facilitate the recruiting process, we provided students with a recruitment ad which articulated the study purpose. Eligible study participants were individuals with fulltime jobs. In the ad, we encouraged participation by offering a monetary incentive; we ensured that participants who completed the three survey waves would receive a compensation of NT $150 (approximately US $5). Students then posted the ad in a number of chat groups in Line. They also asked their peers to help further spread the ad. Interested participants contacted the corresponding author *via* Line by scanning a QR code in the ad, later receiving an informed consent form. In the latter, we again assured them that their participation was voluntary and that their responses would be confidential. We re-approached participants again in one week, as well as 12months after their initial participation. We sent follow-up reminders to participants who did not complete the survey within 2days after receiving it.

Moreover, to ensure data quality, we used participants’ Line IDs to match the three-wave surveys and avoid repetitions. Participants used the virtual confidential IDs they created for themselves without disclosing their real names to the researchers, thus guaranteeing the participants’ anonymity. We also used an attention check strategy (i.e., “For this item, please select 6 and move on to the next item”) to detect and exclude inattentive respondents. After removing mismatched three-wave surveys and careless data, the final sample size was 361, resulting in an overall response rate of 52.24%. The 361 participants filled out three-wave survey questions with no missing data. At T1, the survey was completed by 691 individuals; of these, 578 persons completed the survey again at Time 2 (T2; retention rate of 83.65%). At Time 3 (T3), 361 of these 578 individuals who completed the questionnaire at T1 and T2 completed the final questionnaire.

The 361 participants worked in 9 industry sectors according to the Global Industry Classification Standard (GICS: industrial (3.6%), consumer discretionary (19.6%), consumer staples (2.8%), health care (12.8%), financial (31.3%), information technology (3.9%), communication service (11.1%), utilities (7.8%), and real estate (9.6%). In addition, two-thirds of all participants (66.3%) were female. Participants had an average age of 36.91years (SD=8.89), within the 25-67-year range; their average job tenure was 7.25years (SD=6.57); and 81.40% held a bachelor’s degree. Only 28.80% of participants held a managerial position, and just over half of the sample (54.2%) were married.

We investigated the possibility of selection bias between dropout and final samples by systematically examining differences between participants in the panel sample and the dropouts regarding their demographic data as well as mean scores on the study variables. Our analyses revealed no significant differences for any category. We thus concluded that no significant selection bias had occurred due to panel loss.

### Measures

#### Presenteeism

We assessed presenteeism by using a two-item scale developed by Lu et al. (2013; e.g., “Although you felt sick, you still forced yourself to go to work”). Respondents indicated their agreement with each statement using a 4-point scale (1=never, 2=one to two times, 3=three to four times, 4=more than five times), with higher scores representing more frequent presenteeism. Factor loadings were 0.88–0.91 at T1, 0.91–0.92 at T2, and 0.90–0.92 at T3. Spearman-Brown formula was applied to calculate the reliability of the two-item measurement (Eisinga et al., 2013), the values for the scale were 0.90 (T1), 0.90 (T2), and 0.91 (T3).

#### Effort Exertion

We adopted a ten-item scale developed and validated by De Cooman et al. (2009) to measure three effort exertion factors (persistence, direction, and intensity). Sample items included: “I always work equally hard at my job”; “I do my best to do what is expected of me”; and “I always exert equally hard during the execution of my job.” We used a 7-point Likert scale, ranging from 1 (fully disagree) to 7 (fully agree). We considered effort exertion to be a second-order latent factor in which the items measuring persistence, direction, and intensity were loaded onto their underlying constructs, and these three constructs loaded on the higher-order factor. Fit statistics supported the use of this second-order factor model of effort exertion (root mean square error of approximation (RMSEA)=0.07; comparative fit index (CFI)=0.97; and incremental fit index (IFI)=0.97). Factor loadings were 0.90–0.95 at T1, 0.91–0.94 at T2, and 0.90–0.94 at T3. Cronbach’s α values for the scale were 0.92 (T1), 0.90 (T2), and 0.89 (T3).

#### Emotional Exhaustion

We used a nine-item emotional exhaustion scale based on the Maslach Burnout Inventory (MBI; Maslach et al., 1997). One sample item utilized was “I feel used up at the end of the workday.” We applied a 7-point scale, ranging from 0 (never experience this feeling) to 6 (experience similar feelings every day). Factor loadings were 0.92–0.94 at T1, 0.91–0.93 at T2, and 0.90–0.93 at T3. Cronbach’s α values for the scale were 0.93 (T1), 0.92 (T2), and 0.91(T3).

#### Work Engagement

We used the nine-item Utrecht Work Engagement Scale (UWES-9; Schaufeli et al., 2006) to measure work engagement. Sample items included: “At work, I feel like I am bursting with energy”; “I am enthusiastic about my job”; and “I am immersed in my work.” Scholars have pointed out that the UWES-9 scale is based on a one-dimensional construct but that it encompasses three different but highly correlated concepts and that the corresponding scores indicate overall work engagement (Schaufeli et al., 2006; Bakker and Leiter, 2010). We used a 7-point rating scale, ranging from 1 (never) to 7 (always). Factor loadings were 0.89–0.93 at T1, 0.90–0.93 at T2, and 0.90–0.92 at T3. Cronbach’s α values for the scale were 0.89 (T1), 0.91 (T2), and 0.90 (T3).

#### Job Performance

We applied a four-item scale developed by Ashford et al. (1989) to assess job performance. Items included: “My supervisor is satisfied with my performance” and “I am effective at my job.” We used a 5-point Likert scale, ranging from 1 (disagree very much) to 5 (agree very much). Cronbach’s α values for the scale were 0.87 (T1), 0.89 (T2), and 0.89 (T3).

#### Controls

We controlled for gender (coded male=0, female=1), age, marital status (coded as married=1, not married=0), education level (converted to years of formal education), job tenure (in years), and managerial job position (coded as 1=managers, 0=employees).

### Data Analysis Strategy

To test our research hypotheses, we applied structural equation modelling (*SEM*) techniques (AMOS 22) to examine the direct cross-lagged effects of presenteeism on outcomes over the short (H1) and long terms (H2), as well as testing the mediating role of effort exertion in the presenteeism-outcome relationship over different time spans as proposed in H3 (short-term with a 1-week interval) and H4 (long-term with a 1-year interval). Furthermore, to corroborate the indirect effects of H3 and H4, we also applied bootstrapping methods.

#### Confirmatory Factor Analysis

We first conducted a series of confirmatory factor analyses (*CFA*) to examine the validity of our measures. Our results indicated that the hypothesized 15-factor measurement model (i.e., presenteeism and effort exertion, as well as emotional exhaustion, job performance, and work engagement, etc.) fit the data well: *χ^2^*(*df*=733)=2826.89, *p*<0.001, *CFI*=0.92, *RMSEA*=0.06, and *SRMR*=0.05. All scale items loaded on their intended factors significantly (*p*<0.001). Factor loadings for each item are provided in the Appendix. We then compared the 15-factor model with 105 alternative 14-factor models, where any two of the 15 factors were combined. These results demonstrated that the 15-factor model fit the data significantly better than any other of the 14-factor models (*Δχ^2^*[*Δdf*=14] ranged from 139.66 to 1339.96, *p<* 0.001). Furthermore, they suggested that the measure used in our study captured distinct constructs.

#### Measurement Model and Common Method Variance

To evaluate the extent to which our findings were influenced by common method variance, we followed the procedure recommended by Podsakoff et al. (2003) and widely employed (Ahuja et al., 2007). Following their approach, we estimated three models: (1) Model 1: a null measurement model, (2) Model 2: multifactor measurement model with the proposed latent constructs, and (3) Model 3: measurement model with an additional method factor. If a common method effect existed, Model 3 would fit the data significantly better than Model 2. Then we would need to determine the amount of variance in the model contributed by the single method factor. To do that, we computed the average variance extracted (AVE) for the latent constructs against the method factor. It has been argued that, to rule out the presence of pervasive method variance, the variance explained by the method factor should be less than 25% of the total variance (Williams et al., 1989).

The results demonstrated that: Model 1 [*χ^2^*=3328, *p*<0.001, *χ^2^*/df=27.09, Tucker–Lewis index, TLI=0.56, CFI=0.42, RMSEA=0.17]; Model 2 [*χ^2^*=1112, *p* <0.001, χ^2^/df=2.39, TLI=0.91, CFI=0.91, RMSEA=0.05]; Model 3 [*χ^2^*=2269.11, *p*<0.01, χ^2^/df=21.03, TLI=0.51, CFI=0.61, RMSEA=0.15]. The loadings from model 3 were then used to compute the AVE for each latent construct, including the method factor.

The Model 2 provided a good fit to the data, with RMSEA scores below 0.06, whereas TLI and CFI were above 0.90 (Bentler and Bonnet, 1980). The loadings from model 3 were then used to compute the AVE for each latent construct, including the method factor the method factor accounted for only 14% of the total variance, less than the 25% cut-off recommended by Williams et al. (1989). Thus, we concluded that common method variance did not significantly contaminate the results.

As a supplementary analysis, we also computed alternative models to investigate the causal relations between presenteeism and outcomes. Though we hypothesized that the causal effects of T1 presenteeism on T2 and T3 outcomes were theoretically based, reversed relationships between the constructs are plausible (Lesener et al., 2019). However, the research model, which is composed of M1 and the causal relationships of T1 presenteeism on exhaustion, work engagement, and job performance at T2 &T3 outperformed the stability, reversed causality, and reciprocal models also tested.

#### Longitudinal Factorial Invariance

Examining factorial invariance in this three-wave panel study is important because it helps provide evidence for the imperative assumption that the fundamental meaning of the latent variables is consistent across measurement points. We thus examined invariance by modeling constrained models and comparing all the models to more restricted models. According to our results, the research dimensions were invariant across time by showing intercept invariance [*Δχ^2^* (58)=42.66, *p*=0.59], loading invariance [*Δχ^2^*(46)=36.77*, p*=0.67], configural invariance [*Δχ^2^*(25)=26.88, *p*= 0.36], and residual invariance [*Δχ^2^*(88)=100.24, *p*=0.11].

## Results

### Descriptive Analysis

We report the mean, standard deviations, and correlations among all study variables in Table 1. Most of the relationships between the variables were significant and took the expected directions. Specifically, presenteeism behavior positively correlated with job performance and work engagement at T1 and T2. However, the patterns were diametrically different between T1 and T3. Namely, presenteeism negatively correlated to job performance (−0.18, *p*<0.05) and work engagement (−0.19, *p*<0.05) at T3, but positively correlated to emotional exhaustion at T3 (0.29, *p*<0.001).

**Table 1:** Mean, standard deviations, and correlations among focal research constructs (*N*=361).

	1	2	3	4	5	6	7	8	9	10	11	12	13	14	15
**Time 1**															
1. Presenteeism	(0.90)														
2. Effort exertion	0.21^**^	(0.92)													
3. Work engagement	0.19^*^	0.37^***^	(0.89)												
4. Job performance	0.18^*^	0.36^***^	0.44^***^	(0.87)											
5. Emotional exhaustion	0.11^*^	0.08	−0.13	−0.21^**^	(0.93)										
**Time 2**															
6. Presenteeism	0.62^***^	0.18^*^	0.16^*^	0.26^**^	0.22^*^	(0.90)									
7. Effort exertion	0.31^***^	0.66^***^	0.33^***^	0.33^***^	0.13	0.34^***^	(0.90)								
8. Work engagement	0.16^*^	0.45^***^	0.64^***^	0.20^**^	−0.07	0.18^*^	0.53^***^	(0.91)							
9. Job performance	0.20^**^	0.42^***^	0.41^***^	0.01	0.01	0.22^**^	0.48^***^	0.42^***^	(0.89)						
10 Emotional exhaustion	0.15^*^	0.14^*^	−0.17^*^	0.21^**^	0.52^***^	0.24^**^	0.16^*^	−0.18 ^*^	−0.23^**^	(0.92)					
**Time 3**															
11. Presenteeism	0.53^***^	0.19^*^	−0.12	−0.27^**^	0.37^***^	0.58^***^	0.19^*^	0.03	−0.22^**^	0.34^***^	(0.91)				
12. Effort exertion	0.32^***^	0.45^***^	0.23^**^	−0.22^*^	0.28^**^	0.34^***^	0.63^***^	−0.23^**^	−0.20^*^	0.27^**^	0.25^**^	(0.89)			
13. Work engagement	−0.19^*^	−0.14^*^	0.56^***^	0.13	−0.18^*^	−0.21^*^	−0.14^*^	0.54^***^	0.16	−0.21^*^	0.17^*^	0.32^***^	(0.90)		
14. Job performance	−0.18^*^	−0.18^*^	0.22^**^	−0.20^*^	−0.20^*^	−0.22^*^	−0.18^*^	0.42^***^	0.55^***^	−0.23^*^	0.21^*^	0.38^***^	0.46^***^	(0.89)	
15. Emotional exhaustion	0.29^***^	0.26^***^	0.13^*^	0.26^**^	0.53^***^	0.31^***^	0.31^***^	0.07	0.25^**^	0.63^***^	0.06	0.06	−0.12	−0.28^**^	(0.91)
Mean	5.12	53.10	16.03	47.88	36.72	5.13	53.44	16.88	49.02	37.88	5.27	54.12	15.78	44.31	41.33
SD	1.07	6.91	3.34	3.17	5.13	1.01	6.67	3.12	3.25	5.10	1.23	6.46	3.08	3.08	5.32

* *p<0.05*,

** *p<0.01*,

*** *p<0.001*.

*Cronbach’s α was shown in the diagonal between brackets (two-tailed)*.

### Hypotheses Testing

To test the hypothesized direct and indirect effects, we constructed two *SEM* models corresponding to the different time spans. Thus, every model included presenteeism at T1, effort exertion at T1, and three endogenous variables (job performance, work engagement, and emotional exhaustion) at one week (T2) and one year (T3), respectively.

#### Cross-Lagged Direct Effects of Presenteeism on Outcomes

Hypotheses 1 and 2 examine the short and long-term effects that presenteeism has on outcomes at work. Specifically, hypothesis 1 predicted that displaying presenteeism is conducive to job performance (H1a) and work engagement (H1b), without damaging personal health over a short timeframe.

As reported in Table 2, after controlling for the baseline level of outcomes, the results supported the direct short-term effects of presenteeism on outcomes by corroborating that displaying presenteeism has a positive cross-lagged effect on job performance (*β*=0.13^**^, *p*<0.01) and work engagement (*β*=0.16^**^, *p<* 0.01). Therefore, H1a and H1b are supported.

**Table 2:** Medwiation effects of effort exertion between presenteeism behaviors and outcomes over a 1-month interval (T2) and a 12-month interval (T3).

		*β*	*SE*	*χ^2^/(df)*	*R^2^*	*RMSEA*	*GFI*	*CFI*
**Direct path effects**	
	T1 Pres → Eff T1	0.13^**^	0.04	2349.23/266	0.26	0.07	0.89	0.88
H1a	T1 Pres → Perf T2	0.13^**^	0.01	2823.40/266	0.32	0.07	0.89	0.90
H1b	T1 Pres → Eng T2	0.16^**^	0.02	2543.31/266	0.30	0.06	0.88	0.88
	T1 Pres → Exh T2	0.05	0.02	2399.31/266	0.28	0.08	0.88	0.88
H2a	T1 Pres → Perf T3	−0.24^***^	0.02	2648.42/266	0.29	0.07	0.89	0.90
H2b	T1 Pres → Eng T3	−0.26^***^	0.02	3123.34/266	0.31	0.07	0.89	0.91
H2c	T1 Pres → Exh T3	0.36^***^	0.02	2778.33/266	0.31	0.07	0.88	0.90
**Indirect effects**
H3a	T1 Pres → T1 Eff→Perf T2	0.09^*^	0.02	2923.40/264	0.35	0.05	0.90	0.91
H3b	T1 Pres → T1 Eff→Eng T2	0.10^*^	0.01	2842.46/264	0.33	0.04	0.90	0.91
	T1 Pres → T1 Eff→Exh T2	0.03	0.01	2972.22/264	0.33	0.06	0.89	0.90
H4a	T1 Pres → T1 Eff→Perf T3	−0.17^**^	0.01	2887.21/264	0.33	0.07	0.89	0.90
H4b	T1 Pres → T1 Eff→Eng T3	−0.23^***^	0.02	3347.54/264	0.34	0.07	0.89	0.91
H4c	T1 Pres → T1 Eff→Exh T3	0.16^**^	0.02	3134.23/264	0.35	0.06	0.90	0.91

*N=361. β, standardized path coefficients; SE, standard error; χ^2^/(df)*, *Chi squared (degrees of freedom); RMSEA, Root Mean Square Error of Approximation; GFI, Goodness-of-Fit Index; CFI, Confirmatory Fit Index. T1, Initial test; T2, 1-month interval; T3, 12-month interval. Pres, Presenteeism; Eff*, *Effort exertion; Perf, Performance; Eng, Work engagement; Exh, motional Exhaustion*.

* *p<0.05*,

** *p<0.005*,

*** *p<0.001*.

Although not hypothesized, we also examined the direct effects of presenteeism behavior on emotional exhaustion after a 1-week interval (T2). Our findings do not show any significant positive cross-lagged effects of presenteeism on short-term emotional exhaustion (β=0.05, ns.).

Hypothesis 2 predicted the negative direct effects of presenteeism on job performance (H2a), work engagement (H2b), and the positive direct effect on emotional exhaustion (H2c) over the long-term (T3). As can be seen in Table 2, after controlling for the baseline level of outcomes, our results supported the direct long-term effects of presenteeism on outcomes by corroborating the negative cross-lagged effects on job performance (*β*=−0.24^***^, *p* <0.001), and work engagement *(β*=−0.26^***^, *p* <0.001), as well as a positive cross-lagged effect on emotional exhaustion (*β*=0.36^***^, *p*<0 0.001) at the 12-month interval (T3), thus corroborating hypotheses H2a, H2b, and H2c.

#### The Mediating Effect of Effort Exertion Between Presenteeism and Outcomes

Hypothesis 3 anticipated the mediation effects of effort exertion on the relation between presenteeism and outcomes, namely, displaying presenteeism behavior over the short-term would have positive effects on job performance (H3a) and engagement (H3b) *via* effort exertion. The results in Table 2 indicate that presenteeism has positive cross-lagged relationships with job performance and work engagement *via* effort exertion over a 1-week interval (at T2). Specifically, the indirect effect of presenteeism at T1 on job performance at T2 through effort exertion at T1 was positive (*β*=0.09, *p*<0.05). Therefore, effort exertion mediates the relationship between presenteeism and job performance at T2, supporting H3a.

Likewise, the indirect effect of presenteeism at T1 on work engagement at T2 through effort exertion at T1 was positive (*β*=0.10, *p*<0.05). Thus, effort exertion mediates the relationship between presenteeism and work engagement at T2, supporting H3b. Furthermore, we carried out bootstrap analyses of the mediating effects which further confirmed these results by showing the mediating effect of effort exertion between presenteeism on job performance at T2 (estimate=0.07, *SE*=0.01, 95% *Boot CI*=[0.012; 0.019]) and work engagement at T2 (estimate=0.08; *SE*=0.02; 95% *Boot CI*=[0.023, 0.039]), as evidenced by a 95% bias-corrected bootstrap confidence interval that did not include zero. Therefore, the H3a and H3b were supported.

Hypothesis 4 predicted that exhibiting presenteeism behavior over the long-term would hinder job performance (H4a) and work engagement (H4b), while provoking greater emotional exhaustion (H4c) *via* effort exertion. Our results in Table 2 indicate that presenteeism has negative cross-lagged relationships with job performance and work engagement as well as positive cross-lagged relationship with emotional exhaustion *via* effort exertion over a 1-year interval (T3). Specifically, the indirect effect of presenteeism at T1 on job performance at T3 through effort exertion at T1 was negative (*β*=−0.17, *p*<0.01). Therefore, effort exertion mediates the relationship between presenteeism at T1 and job performance at T3, supporting H4a.

Likewise, the indirect effect of presenteeism at T1 on work engagement at T3 through effort exertion at T1 was negative (*β*=−0.23, *p*<0.001). Thus, effort exertion mediates the relationship between presenteeism at T1 and work engagement at T3, supporting H4b. Similarly, the indirect effect of presenteeism at T1 on emotional exhaustion at T3 through effort exertion at T1 was positive (*β*=0.16, *p*<0.01) in the long run (T3). In sum, effort exertion at T1 mediated the relationship between presenteeism at T1 and emotional exhaustion at T3, supporting H4c.

Moreover, our bootstrap analyses of the mediating effects further confirmed these results by showing the mediating effect of effort exertion between presenteeism on job performance (estimate=−0.08, *SE*=0.01, 95% *Boot CI*=[−0.014; −0.009]) and work engagement (estimate=−0.09, *SE*=0.02, 95% *Boot CI*=[−0.012; −0.008]), as well as emotional exhaustion (estimate=0.12, *SE*=0.01, 95% *Boot CI*=[0.001; 0.017]) at the 12-month interval (T3). Therefore, the H4a, H4b, and H4c were supported.

Overall, our findings support all the proposed hypotheses and raise important issues concerning the management of presenteeism. Furthermore, our proposed process-based model of presenteeism highlights and captures the differential effect of presenteeism behaviors across different time spans.

## Discussion

Our study answers the call to provide empirical evidence regarding the differential effects of timeframes on employee presenteeism patterns (Lohaus and Habermann, 2019). This pioneering research helps understand the psychological mechanisms influencing presenteeism over distinct timeframes (one week and one year), each leading to different personal and work outcomes, whether positive or negative.

The aim of this study was two-fold: shed light on the inconsistent results of previous presenteeism studies by investigating its short and long-term effects and understand the psychological mechanisms transforming neutral presenteeism behavior into positive or negative outcomes. In accordance with recent cross-discipline reviews (Ruhle et al., 2020), we consider effort exertion to be the underlying mechanism for presenteeism’s effects. We test a process-based model, namely, the presenteeism–effort exertion–outcomes linkage, to analyze the relationship between overt presenteeism behavior and distinct outcomes. Our findings strongly corroborate this model, which differentiates short and long-term presenteeism (H1 and H2) and captures the process-based model of presenteeism (H3 and H4).

These results provide strong evidence of the distinctive outputs of short versus long-term presenteeism. Indeed, working while feeling unwell for a short period of time is conducive to job performance (H1a) and work engagement (H1b), without negatively affecting personal well-being. However, recurrent presenteeism and overwork lead to decreased job performance (H2a), work engagement (H2b), and well-being (H2c) after a year. Furthermore, these relationships are mediated by effort exertion. Specifically, we found presenteeism to be positively related to job performance and work engagement through effort exertion over a one-week interval (H3a and H3b). However, the same scenario generated distinct patterns over time. Through effort exertion, presenteeism was negatively related to job performance and work engagement, though positively related to emotional exhaustion over a one-year interval (H4a–c). These effects were attributed to effort exertion, the psychological mechanism underlying observable presenteeism behavior.

By differentiating between overt presenteeism behavior and real effort exerted through presenteeism, our multi-timeframe study reconciles the contradictory consequences of presenteeism behaviors found in previous research. Some scholars have described presenteeism as exerting effort at work despite exhaustion or illness, indicating high engagement with a task and, therefore, beneficial over the short-term (Biron and Saksvik, 2009; Godøy, 2016; Karanika-Murray and Biron, 2020). However, presenteeism can represent a silent cost for organizations over longer periods of time (e.g., Demerouti et al., 2009; Gustafsson and Marklund, 2014; Lu and Chou, 2020). The reason for this remarkable change has not been explained in prior research. By considering effort exertion, we connect stress theory with resource theory to address this research gap. In accordance with previous studies that identified effort exertion as an invisible mechanism regulating individual efforts and work performance (e.g., Brown and Peterson, 1994; Brown and Leigh, 1996; Cook et al., 2000), our findings measuring presenteeism over different timeframes clarify how individuals allocate their limited effort capacity to work activities and how this allocation leads to different outcomes. Overall, by incorporating time into a process-based model, our framework offers a more nuanced treatment of presenteeism.

### Theoretical Contribution

From a theoretical perspective, our study has important implications for the presenteeism research field. Different theories have been adopted to explain the inconsistent presenteeism outcomes for different time periods, though an overarching theory to elucidate the consequences of presenteeism has been lacking. For example, job demand-resource theory (Bakker and Demerouti, 2007) is commonly applied in longitudinal research to explain the negative consequences of presenteeism on job performance and well-being (e.g., Baker-McClearn et al., 2010; Deery et al., 2014; McGregor et al., 2016). By contrast, cross-sectional studies have adopted self -determination theory (Deci and Ryan, 2000) to explain the relationship between presenteeism and positive work-related outcomes (e.g., Cooper and Lu, 2019; Karanika-Murray and Biron, 2020). Thus, we provide a comprehensive conceptual model to explain the paradoxical outcomes of presenteeism behavior.

By considering the temporal effects of the stressor and by using the CATS and COR frameworks, we attempt to harmonize these findings. Thus, our results reveal how the presenteeism–effort exertion–outcomes linkage performs over different time spans. Being able to use a single model to explain prior contradictory presenteeism results will benefit the presenteeism field with a more systematic research design and robust findings.

Our proposed process-based model of presenteeism parallels the effort–recovery model (E-R model; (Meijman and Mulder, 2013), which suggests that effort activates “load reactions” leading to physiological and psychological reactions. These reactions are normal responses among people coping with demands over a short timeframe; however, without sufficient recovery time, load reactions have detrimental effects on job performance (Williams et al., 2006; ten Brummelhuis and Bakker, 2012) and health (Hall et al., 2010). Both CATS and E-R theories consider work demands as neutral stimuli within an expected duration. They highlight time relevance by suggesting that prolonged overwork and the resulting effort to meet the demands of work can harm well-being and performance.

Our study extends this research by demonstrating a mechanism through which presenteeism leads to short-term positive work-related outcomes but to impaired well-being and performance over the long-term. Our findings corroborate recovery literature. Research applying the E-R model has found promising effects of different forms of recovery on future work-related outcomes such as job performance and well-being (Sonnentag and Fritz, 2015). Some scholars have documented the beneficial effects of taking time off during non-work time (e.g., not thinking about work after regular working hours) on future personal well-being and organizational outcomes. For example, Lu and Chou (2020) found that psychological detachment during off-job time can act as a buffer in decreasing the lasting negative effects of heavy workloads on work engagement as well as job performance. In addition, in a five-wave follow-up study, Meier and Cho (2019) found that switching-off psychologically after work can decrease the negative impact of work stressors on family relationships, in particular, the relationship with partners. Therefore, future research could include recovery in presenteeism research to clarify the role of rest time in coping with demanding work environments.

### Managerial Implications

The results of our study also have implications for both organizational strategies and managerial practice. As we have corroborated, attending work while unwell has a catalytic effect on effort exertion, engagement, and performance, as well as on increased productivity for an organization though only in the short-term. These results may be more salient due to the Chinese cultural background of our sample. Lu and Chou (2017) have applied social cognitive theory (Bandura, 1997) to explain Chinese self-efficacy in displaying presenteeism behavior. The internalized Confucian cultural norms regarding both hard work and diligence as well as the social relatedness and interpersonal harmony of Chinese employees may function as push-and-pull factors for personal decisions regarding time commitments at work. However, such behavior harms employee performance and well-being over time. Therefore, organizations must attempt to decrease these practices to prevent the “accumulative consequences on downstream health” (Johns, 2010, p. 533).

Organizations and managers should clarify and ensure that taking sick leave when necessary is allowed and duly adjust task allocation or find replacements to reduce pressure on employees to adopt presenteeism behaviors. The appropriate use of sick leave and recovery as a health-promoting strategy is well-documented in recovery research (Bakker and Demerouti, 2017). Moreover, organizations should create work environments in which attractive incentives or other extrinsic reinforcements (e.g., praise) are provided in order to increase employee perceptions of fairness and organizational support (Olafsen et al., 2015). Both psychological states improve employee well-being (Eisenberger et al., 1999).

Furthermore, a central idea of our study is that, in order to obtain long-term sustainability, organizations should tend to employees’ health and review corporate health management policies to ensure that, wherever possible, they do not penalize staff who take sick leave for legitimated reasons. This viewpoint provides implications for organizations and individuals given current trends regarding the adoption of digital practices and the increased virtualization of work, processes which the COVID-19 pandemic has accelerated.

Thereby, employees may also continue to work during illness or return to work too soon, as they do not want to let down their managers and colleagues because they believe that their fellow employees do not consider them sufficiently unwell to take time off (Hayes et al., 2020). Research regarding the impacts of presenteeism by employees with infectious conditions has found that people frequently continue to work while experiencing contagious flu-like symptoms, raising particularly serious public health concerns given the current pandemic (Webster et al., 2019). Therefore, organizational practices should consider that those who have contracted a fairly “mild” case of COVID-19 might return to work while experiencing symptoms such as chronic fatigue and cognitive difficulties several months later; this, in turn, could damage individual well-being, vigor, and organizational effectiveness over the long-term.

### Limitations and Directions for Future Research

Our study has some limitations, though each also represents an opportunity for future research. First, we used self-reported measures, which may suffer from common method variance bias (Podsakoff et al., 2003). To minimize this, we adopted a longitudinal research design to separate the explanatory variables (presenteeism and effort exertion) over time from dependent variables (job performance, work engagement, and well-being). In addition, we conducted a *post hoc* analysis using Harman’s single-factor test (Podsakoff et al., 2003) to detect any possible effects. However, future research could adopt a supervisor–employee dyadic study design to cross-validate our findings, including job performance ratings by supervisors.

We also extended existing studies on presenteeism to a non-Western society; however, the generalization of our findings may be limited by the convenience sample we recruited in Taiwan. Future studies should recruit larger and more representative samples to allow for the generalizability of our research findings in both Western and Eastern contexts.

Additionally, there is evidence that the type of health conditions predispose a person to work while ill (Gosselin et al., 2013); therefore, future research could include employees’ specific health status as a control variable. Similarly, we did not control the firm-level contextual characteristics, which may result in omitted variable bias. However, we did control for some organizational factors such as organizational pressure to work and job replacement policies (not reported in the tables as they were nonsignificant). Future research could also pursue studies in different industries and countries to capture cross-cultural values and additional contextual characteristics.

## Conclusion

This study contributes to shed light on the presenteeism behaviors which have a differential effect based on the scope considered (short versus long-term). By reconciling inconsistent findings regarding the outcomes of presenteeism behavior thanks to our study’s robust design, our research thus offers a more neutral perspective for the prevailing negativist view of presenteeism behavior. This study extends the organizational presenteeism research domain by clarifying the relationships between presenteeism and its outcomes, as well as corroborating that effort exertion mediates the relationship between presenteeism and work-related outcomes.

## Data Availability Statement

The raw data supporting the conclusions of this article will be made available by the authors, without undue reservation.

## Author Contributions

C-YC: conceptualization, funding acquisition, formal analysis, and resources. C-YC and MM: methodology, writing—original draft preparation, and writing – review. MM: editing and finalize. All authors have read and agreed to the published version of the manuscript.

## Funding

This research was funded by a grant from the Ministry of Science and Technology, Taiwan, MOST 109-2410-H-035-048.

## Conflict of Interest

The authors declare that the research was conducted in the absence of any commercial or financial relationships that could be construed as a potential conflict of interest.

## Publisher’s Note

All claims expressed in this article are solely those of the authors and do not necessarily represent those of their affiliated organizations, or those of the publisher, the editors and the reviewers. Any product that may be evaluated in this article, or claim that may be made by its manufacturer, is not guaranteed or endorsed by the publisher.
